# Multilayered regulation of longevity in *Caenorhabditis elegans*

**DOI:** 10.1016/j.mocell.2025.100308

**Published:** 2025-12-11

**Authors:** Dajeong Bong, Hyunwoo C. Kwon, Seung-Jae V. Lee

**Affiliations:** Department of Biological Sciences, Korea Advanced Institute of Science and Technology, 291 Daehak-ro, Yuseong-gu, Daejeon 34141, South Korea

**Keywords:** *Caenorhabditis elegans*, Dietary restriction, Germline deficiency, Insulin/insulin-like growth factor 1 signaling, Longevity, Mild mitochondrial inhibition

## Abstract

Aging in *Caenorhabditis elegans* is regulated by evolutionarily conserved pathways that coordinate cellular maintenance and systemic homeostasis. Here, we review recent advances on four major longevity regimens, including reduced insulin/insulin-like growth factor 1 signaling (IIS), dietary restriction (DR), mild inhibition of mitochondrial respiration, and germline deficiency. Each longevity-promoting regimen enhances protein and RNA quality control, metabolic remodeling, and stress resistance to delay functional declines with age. Reduced IIS strengthens proteostasis and RNA surveillance. DR remodels metabolism and activates autophagy. Mild mitochondrial inhibition elicits adaptive redox signaling and quality control responses. Germline deficiency links reproductive cues to somatic maintenance. We highlight that longevity arises from the integrated regulation of transcriptional, metabolic, and inter-tissue signaling networks. Our review will provide valuable insights obtained from *C. elegans* into the conserved mechanisms of aging, facilitating the development of interventions that promote healthy longevity in humans.

## Graphical Abstract

**Figure:**
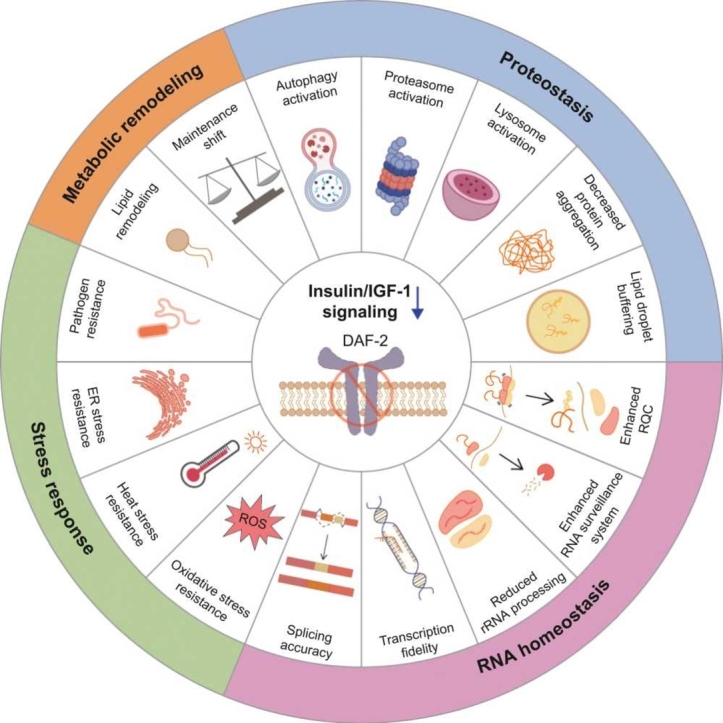

## INTRODUCTION

Aging is a universal biological process characterized by a gradual decline in molecular, cellular, and physiological integrity, leading to impaired function, increased disease susceptibility, and mortality (Ha and Lee, 2023; Ham and Lee, 2020; Hwang et al., 2025; Kang et al, 2024; Kim and Lee, 2019; Kim et al., 2021; Kim et al., 2022; Kwon et al., 2023; Lee et al., 2015; Lee et al., 2021a; López-Otín et al., 2023; Melzer et al., 2020; Park et al., 2024; Son et al., 2019; Yan et al., 2024). The nematode *Caenorhabditis elegans* is a powerful model organism for aging research because of its short lifespan, well-annotated genetics, and conservation of signaling and metabolic pathways across species (Kang et al., 2025; Kim et al., 2020a; Kwon and Lee, 2025; Kwon et al., 2018; Kwon et al., 2023; Lee and Lee, 2022; Lee et al., 2021a; Park et al., 2017). Genetic and molecular studies in *C. elegans* have defined fundamental mechanisms that regulate longevity and established frameworks for understanding aging in complex organisms, providing the possibility for the development of therapeutics for healthy aging (An et al., 2017; Campisi et al., 2019; Hwang and Lee, 2011; Hwang et al., 2012; Jeong et al., 2012; Kenyon, 2010; Lapierre and Hansen, 2012; Lee et al., 2017; Son et al., 2019).

**Fig. 1:**
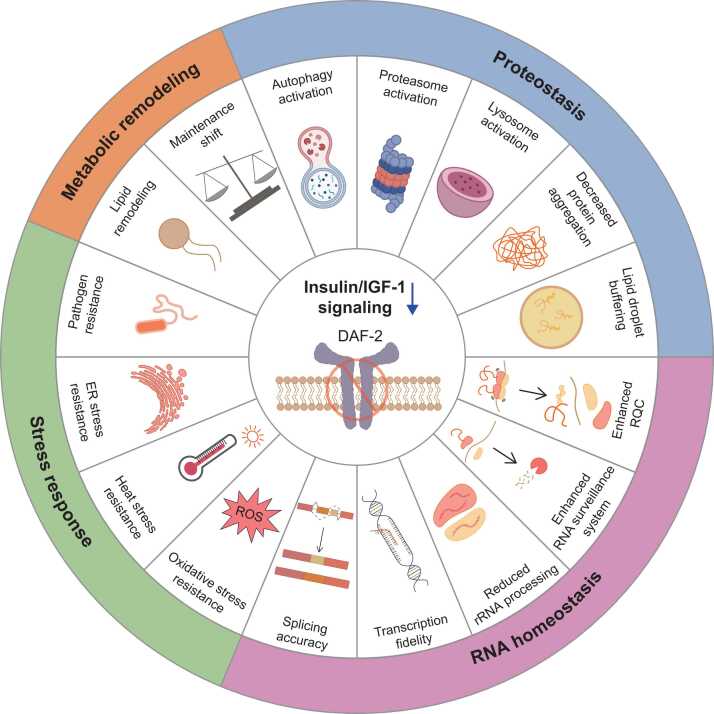
Reduced insulin/insulin-like growth factor 1 (IGF-1) signaling (IIS) promotes longevity in *Caenorhabditis elegans.* C. elegans with reduced IIS, exemplified with genetic inhibition of DAF-2/Insulin/IGF-1 receptor, exhibits robust lifespan extension. Reduced IIS reorganizes cellular processes through multiple domains of longevity regulation, including metabolic remodeling, proteostasis, RNA homeostasis, and stress response. Metabolic remodeling under reduced IIS includes lipid remodeling and a maintenance shift, indicating a transition toward energy conservation. Proteostasis is enhanced through autophagy activation, proteasome activation, lysosome activation, decreased protein aggregation, and lipid droplet buffering, collectively supporting the maintenance of protein quality control. RNA homeostasis is improved by RNA-regulatory pathways, including enhanced ribosome-associated quality control (RQC) and RNA surveillance system, reduced rRNA processing, preserved transcription fidelity, and splicing accuracy, thereby limiting aberrant transcript accumulation and preserving RNA quality during aging. Reduced IIS increases oxidative stress resistance to reactive oxygen species (ROS), heat stress resistance, endoplasmic reticulum (ER) stress resistance, and pathogen resistance.

**Fig. 2:**
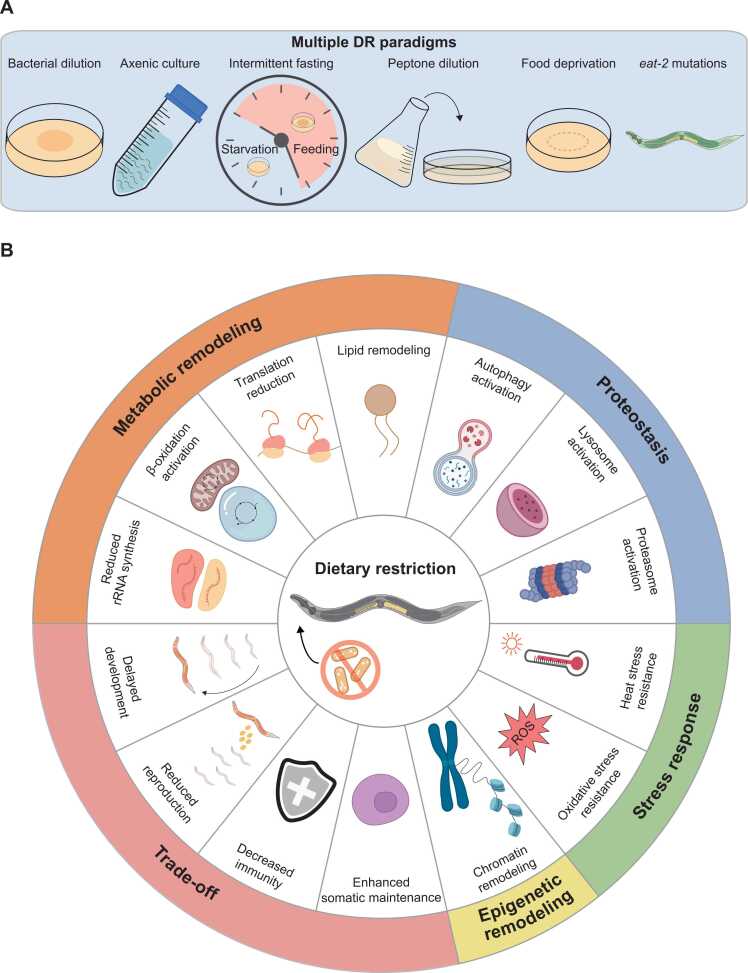
Dietary restriction (DR) increases lifespan in *C. elegans.* (A) Multiple DR paradigms, including bacterial dilution, axenic culture, intermittent fasting, peptone dilution, bacterial food deprivation, and the genetic DR-mimetic *eat-2* mutations, trigger both shared and distinct nutrient-sensing, metabolic, and transcriptional responses. (B) DR remodels multiple biological processes, including metabolic remodeling, proteostasis, stress response, epigenetic remodeling, and trade-off. DR remodels cellular metabolism through reduced rRNA synthesis, β-oxidation activation, translation reduction, and lipid remodeling, indicating a shift toward energy conservation and reallocation of resources. Proteostasis is enhanced by autophagy activation, lysosome activation, and proteasome activation, which promote degradation of damaged components. DR increases oxidative stress resistance to ROS and heat stress resistance. Epigenetic remodeling includes chromatin remodeling and other chromatin-based regulatory adaptations that regulate gene expression programs under DR. DR also involves characteristic trade-offs, such as delayed development, reduced reproduction, and decreased immunity, while simultaneously enhancing somatic maintenance. These opposing changes indicate a shift in resources from growth and reproduction to cellular maintenance.

**Fig. 3:**
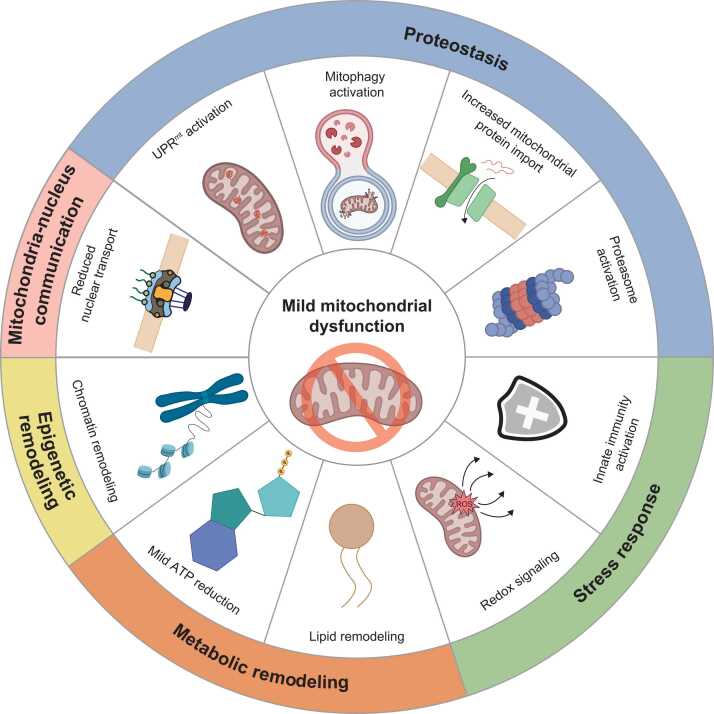
Mild mitochondrial dysfunction lengthens lifespan in *C. elegans.* Mild inhibition of mitochondrial respiration induces downstream responses that remodel cellular processes, including proteostasis, stress response, metabolic remodeling, epigenetic remodeling, and mitochondrial-nucleus communication. Proteostasis is enhanced by mitochondrial unfolded protein response (UPR^mt^) activation, mitophagy activation, increased mitochondrial protein import, and proteasome activation to remove damaged mitochondrial components and to maintain mitochondrial protein quality. Mild mitochondrial dysfunction promotes modest ROS-mediated redox signaling, which activates innate immunity and longevity. Metabolic remodeling includes mild ATP reduction and lipid remodeling, which shift metabolic flux toward maintenance and stress adaptation. Chromatin remodeling drives epigenetic remodeling by reprogramming transcriptional states. Mild mitochondrial dysfunction reduces nuclear pore complex transport, thereby coupling mitochondria-to-nucleus communication.

**Fig. 4:**
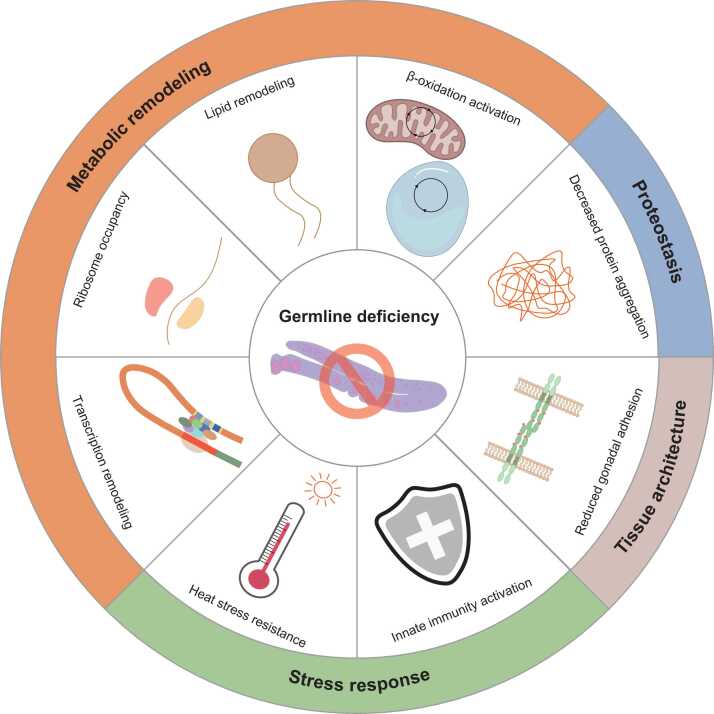
Germline deficiency promotes longevity in *C. elegans.* Germline deficiency reprograms cellular processes, including proteostasis, tissue architecture change, stress response, and metabolic remodeling. Proteostasis is enhanced through decreasing protein aggregation. Germline deficiency alters tissue architecture through transcription remodeling and reduced gonadal adhesion. Germline deficiency also increases innate immunity and heat stress resistance. Metabolic remodeling involves transcription remodeling, ribosome occupancy, lipid remodeling, and β-oxidation activation, indicating a shift in metabolic resources from reproduction to somatic maintenance.

Aging regulation in *C. elegans* is mediated by several evolutionarily conserved signaling pathways. The insulin/insulin-like growth factor 1 (IGF-1) signaling (IIS) pathway is an important regulator of longevity. Reduction in IIS, such as genetic inhibition of *daf-2*, which encodes the insulin/IGF-1 receptor, dramatically extends lifespan (Kenyon et al., 1993, Kimura et al., 1997). The IIS acts through conserved transcription factors that regulate genes that affect stress resistance, proteostasis, and metabolism (Altintas et al., 2016; Kenyon, 2010; Lee and Lee, 2022; Lee et al., 2015b) (Fig. 1). Reduced IIS maintains proteostasis through autophagy and lysosomal activity, and enhances RNA surveillance systems such as nonsense-mediated mRNA decay (NMD) and ribosome-associated quality control (RQC), thereby delaying functional declines during aging (Kwon et al., 2023; Lee and Lee, 2022; Son and Lee, 2017). Dietary restriction (DR), defined as reduced nutrient intake without malnutrition, is another major longevity regimen conserved from yeast to mammals (Fontana et al., 2010, Green et al., 2022). In *C. elegans*, various DR paradigms extend lifespan through overlapping but not identical molecular mechanisms. These pathways inhibit biosynthetic processes, activate autophagy, and promote metabolic adaptation and stress resistance (Fontana et al., 2010; Green et al., 2022; Lee et al., 2015a; Lee et al., 2015b) (Fig. 2). Inhibition of mitochondrial respiration lengthens lifespan through adaptive metabolic and stress signaling. Mild impairment of mitochondrial electron transport chain components decreases respiration but increases lifespan (Hwang et al., 2012, Lee et al., 2015b). The mild inhibition of mitochondrial respiration promotes longevity by moderately increasing reactive oxygen species (ROS) levels, which activate stress response pathways (Hwang et al., 2012, Lee et al., 2015b). The longevity pathways triggered by inhibition of mitochondrial respiration involve the mitochondrial unfolded protein response (UPR^mt^), chromatin remodeling, and mitophagy-dependent organelle clearance, which together maintain mitochondrial and cellular homeostasis (Hwang et al., 2012; Guo and Chiang, 2022) (Fig. 3). The reproductive system also affects organismal aging through communication with somatic tissues (Antebi, 2013, Lee et al., 2015b). The somatic gonad in germline-deficient animals promotes longevity by regulating steroid signaling, DAF-16/Forkhead box O (FOXO) activity, and fat metabolism (Lee et al., 2015b) (Fig. 4). These signals enhance somatic maintenance and contribute to extended lifespan (Kenyon, 2010, Lee et al., 2015b).

Here, we review recent progress in studies regarding the regulation of aging and longevity in *C. elegans*, focusing on four major conserved pathways: reduced IIS, DR, inhibition of mitochondrial respiration, and germline deficiency. We discuss how these longevity-regulating pathways modulate key cellular maintenance systems, including protein and RNA homeostasis, as well as stress resistance, metabolism, and organelle quality control. We also highlight newly identified factors that contribute to these signaling pathways and expand the molecular framework of aging regulation. Building upon our previous overview of *C. elegans* longevity pathways (Lee et al., 2015b), this review highlights major advances over the past decade and integrates them into a coherent view of how conserved pathways regulate aging and longevity. We comprehensively discuss multilayer regulation to indicate the hierarchically integrated framework that connects the four major longevity paradigms with their downstream mechanistic pathways and the regulatory hierarchy that spans molecular, cellular, and organismal levels. Overall, this review will provide a recent understanding of the conserved mechanisms regulating lifespan in *C. elegans* and their implication in elucidating human aging, which may help developing strategies to promote healthy longevity.

## MAIN BODY

### Mechanisms of Longevity Regulation Under Reduced IIS in *C. elegans*

Reduction of IIS drastically extends lifespan in *C. elegans* (Kenyon et al., 1993, Kimura et al., 1997). The role of this longevity regimen is conserved across species from *C. elegans* and *Drosophila melanogaster* to mice and humans (Blüher et al., 2003; Fontana et al., 2010; Holzenberger et al., 2003; Kenyon et al., 1993; Kojima et al., 2004; Suh et al., 2008; Tatar et al., 2001). Reduced IIS extends lifespan by activating downstream transcription factors, including DAF-16/FOXO, HSF-1/heat shock factor 1 (HSF1), SKN-1/nuclear factor erythroid 2-related factor (NRF), and HLH-30/transcription factor EB (TFEB) (Altintas et al., 2016; Kenyon, 2010; Lee and Lee, 2022; Nakamura and Yoshimori, 2018). These transcription factors regulate a broad set of genes involved in proteostasis, RNA homeostasis, stress resistance, metabolism, and innate immunity, collectively producing profound effects on organismal longevity (Altintas et al., 2016; Kenyon, 2010; Lee and Lee, 2022) (Fig. 1).

Proteostasis is a central maintenance process regulated by IIS. In *daf-2* mutants, proteostasis is enhanced through multiple coordinated mechanisms. Autophagic flux gradually decreases with age in the intestine, body wall muscles, pharynx, and neurons, but *daf-2* mutants preserve higher autophagic flux across these tissues, suggesting that reduced IIS maintains proteostasis by sustaining autophagic activity (Chang et al., 2017). The myogenic transcription factor UNC-120/serum response factor (SRF) maintains reduced autophagosome accumulation in the muscles of *daf-2* mutants compared with wild-type (WT) animals (Mergoud Dit Lamarche et al., 2018). In addition, age-associated lysosomal dysfunction is attenuated in *daf-2* mutants, promoting protein aggregate clearance in a DAF-16/FOXO- and SKN-1/NRF-dependent manner (Sun et al., 2020). Activation of SKN-1A isoform, the homolog of NRF1, by PNG-1/N-glycanase 1 (NGLY1)-dependent protein sequence editing is essential for upregulation of proteasome genes and the maintenance of proteostasis (Lehrbach et al., 2019). Consistently, *daf-2* mutants exhibit reduced protein aggregation and paralysis in models of proteotoxic diseases such as polyglutamine (polyQ), amyloid-β (Aβ), and α-synuclein, highlighting that IIS reduction enhances proteostasis across diverse proteotoxic contexts (Florez-McClure et al., 2007; Haque et al., 2020; Jo et al., 2023; Moronetti Mazzeo et al., 2012). In *daf-2* mutants, intestinal autophagy receptor SQST-1/sequestosome 1 (SQSTM1/p62) accumulation is reduced compared with WT, supporting their enhanced proteostasis (Kumar et al., 2023). While moderate SQST-1/SQSTM1/p62 activation promotes autophagy and longevity, hyperactivation compromises proteostasis, and lipid droplets contribute to alleviating proteotoxic stress (Kumsta et al., 2019, Kumar et al., 2023). Late-life degradation of DAF-2 is sufficient to reactivate proteostasis networks, clearing age-dependent aggregates of endogenous proteins, such as PAB-1/poly(A)-binding protein (PABP1) and lipid-binding protein 2 (LBP-2), and restoring stress resilience to youthful levels (Molière et al., 2024). Proteomic analysis demonstrates that *daf-2* mutations increase the levels of stress-response proteins and decrease the levels of metabolic proteins, reflecting a systematic shift toward maintenance and protection (Stout et al., 2013). In *daf-2* mutants, most proteins exhibit age-dependent changes at rates similar to WT animals, while selectively sustaining the levels of proteins associated with the proteasome, chaperones, and stress-response factors (Narayan et al., 2016). Reduced IIS remodels protein turnover dynamics with age. *daf-2* mutants exhibit reduced protein synthesis and degradation during early adulthood, maintaining proteostasis not by accelerating turnover but by stabilizing proteins through trehalose, which enhances protein solubility and prevents aggregation (Depuydt et al., 2016). Ubiquitin-proteome profiling showed that *daf-2* mutations preserve ubiquitination of proteasomal targets during aging (Koyuncu et al., 2021). These data suggest an adaptive strategy that helps conserve resources initially and then reinforce proteostasis as damage accumulates.

RNA quality control is enhanced under reduced IIS. In particular, NMD, a key mRNA surveillance mechanism to eliminate premature termination codon (PTC)-containing mRNA transcripts (Boo et al., 2024; Kim and Maquat, 2019; Li et al., 2024c; Shin et al., 2024), is upregulated in *daf-2* mutants (Kim et al., 2020b; Son and Lee, 2017; Son et al., 2017). Transcriptome analysis indicates that the levels of endogenous NMD targets, including PTC- and upstream open reading frame (uORF)-containing transcripts, are decreased in *daf-2* mutants (Son et al., 2017). The ATP-dependent RNA helicase suppressor with morphological effect on genitalia (SMG)-2/up-frameshift 1 (UPF1), a core component of the NMD pathway, is essential for several longevity regimens including reduced IIS (Son et al., 2017). Multiple components of the NMD pathway, including SMG-1/SMG1 through SMG-5/SMG5, are required for the longevity of *daf-2* mutants, with neuronal NMD playing a crucial role. In addition, the positive NMD regulator ALGN-2/alpha-1,3/1,6-mannosyltransferase (ALG2), whose level increases in *daf-2* mutants, is essential for the extended lifespan (Kim et al., 2020b). Thus, ALGN-2/ALG2-dependent enhancement of NMD contributes to longevity conferred by reduced IIS. Furthermore, combinatorial transcriptomic and genetic analyses show that the NMD factor SMG-2/UPF1 functionally interacts with the autophagy regulator HLH-30/TFEB to promote longevity in *daf-2* mutants, showing that reduced IIS coordinates RNA and protein quality control systems for exerting anti-aging effects (Ham et al., 2024). Ribosome-associated surveillance pathways, including RQC, also contribute to maintaining RNA quality during aging (Kwon et al., 2023; Lee et al., 2025; Stein et al., 2022). Ribosome stalling on aberrant mRNA increases with age, while the activity of RQC, which resolves stalled ribosomes and promotes the degradation of aberrant mRNAs and proteins, decreases in aged *C. elegans* (Stein et al., 2022, Lee et al., 2025). In *daf-2* mutants, however, RQC activity and associated mRNA surveillance mechanisms such as no-go decay (NGD) resolving ribosome stalling on structured or rare-codon regions and nonstop decay (NSD) targeting stop codon-lacking transcripts are maintained at elevated levels (Lee et al., 2025). The ribosome rescue factor PELO-1/pelota (PELO)-dependent RQC contributes to longevity in *daf-2* mutants by preventing the accumulation of faulty translation products and aberrant transcripts (Lee et al., 2025). Reduced IIS also remodels RNA metabolism at the transcriptional level. The RNA helicase HEL-1/DExD-box helicase 39A (DDX39A) acts as a transcriptional co-regulator that increases DAF-16/FOXO activity and is required for the longevity of *daf-2* mutants (Seo et al., 2015). The other RNA helicase SACY-1/DEAD-box helicase 41 (DDX41) is also required for the long lifespan of *daf-2* mutants without affecting the transcriptional activity of DAF-16/FOXO (Seo et al., 2016). Systematic transcriptome analyses show that *daf-2* mutations delay physiological aging by preserving transcriptional fidelity and decreasing age-dependent increases in alternative 3′ splice site usages (Ham et al., 2022). Collectively, these findings highlight that reduced IIS contributes to longevity through both improved RNA surveillance and remodeled RNA metabolism.

Reduced IIS increases stress resistance, contributing to longevity. *daf-2* mutations increase resistance to oxidative and heat stresses in a DAF-16/FOXO-dependent manner (Lee and Lee, 2022; Lee et al., 2015b). Intrinsic thermotolerance in *daf-2* mutants also depends on *de novo* protein translation of DAF-16/FOXO target genes such as *ctsa-3.2*/cathepsin A (*CTSA*), a lysosomal serine carboxypeptidase (McColl et al., 2010). SKN-1/NRF also contributes to enhanced oxidative stress resistance and longevity in *daf-2* mutants, as reduced IIS promotes SKN-1/NRF nuclear localization and upregulation of detoxification genes (Tullet et al., 2008). HSF-1/HSF1 cooperates with DAF-16/FOXO to enhance thermotolerance in *daf-2* mutants by upregulating small heat shock protein genes, preventing protein aggregation, and promoting longevity (Hsu et al., 2003; Morley and Morimoto, 2004; Murphy et al., 2003). The enhanced stress resistance also requires the endosomal trafficking protein TBC-2/TBC1 domain family member 2 (TBC1D2), which is necessary for proper DAF-16/FOXO subcellular localization, linking vesicle trafficking to reduced IIS-mediated stress adaptation (Traa et al., 2023). DAF-16/FOXO and HSF-1/HSF1 also increase immunity in aged *daf-2* mutants by decreasing the expression of *zip-10*/basic leucine zipper transcription factor (*bZIP*), which in turn decreases the level of an agonistic insulin-like peptide (ILP), INS-7 (Lee et al., 2021b). In addition, *daf-2* mutations reduce lethal pharyngeal infection through increasing pharyngeal DAF-16/FOXO activity (Zhao et al., 2021). These findings demonstrate that IIS reduction coordinates stress resistance and innate immunity to delay aging.

Beyond the intrinsic effects of *daf-2* mutations, additional chemical and genetic modulators of IIS further diversify the mechanisms of longevity regulation. Small molecules, including carbamazepine (a voltage-gated channel inhibitor) and calmagite (a calcium and magnesium indicator), increase nuclear translocation of DAF-16/FOXO and upregulate downstream stress-responsive genes, promoting metal stress resistance and increasing healthspan (Ye et al., 2025). Similarly, paeonol reduces IIS and enhances stress resistance under oxidative and thermal stress conditions (Li et al., 2024b). Mutations in *chn-1*/carboxyl-terminus of Hsc-70-interacting protein (*CHIP*), which increase the stability of DAF-2 receptor, reduce lifespan in WT animals by increasing IIS activity in adults (Falsztyn et al., 2025; Tawo et al., 2017). Hypomorphic mutations in *daf-18*/phosphatase and tensin homolog (*PTEN*), which encodes a key activator of DAF-16/FOXO, maintain the extended lifespan of *daf-2* mutants while ameliorating their development and motility defects (Park et al., 2021a). Mechanistically, the hypomorphic mutations in *daf-18*/*PTEN* retain partial DAF-16/FOXO activity and prevent hyperactivation of SKN-1/NRF (Park et al., 2021a). In addition to molecular regulators of IIS, tissue-level coordination of IIS plays a key role in systemic longevity regulation by integrating neuronal inputs with organismal signaling networks (Altintas et al., 2016; Jeong et al., 2012; Kenyon, 2010; Kim et al., 2020a). Neuronal IIS activation substantially suppresses longevity in *daf-2* mutants, whereas restoring IIS in muscle or the intestine has relatively small effect on their longevity (Wolkow et al., 2000). This finding establishes the nervous system as the primary tissue for reduced IIS-mediated longevity in *C. elegans*. Reduced neural excitation promotes longevity by activating DAF-16/FOXO through *C. elegans* RE1-silencing transcription factor (REST) orthologs, suppressor of presenilin defect (SPR)-3 and -4 (Zullo et al., 2019). Genetic inhibition of *spr-3* and *spr-4* increases neural excitation and suppresses the extended lifespan of *daf-2* mutants (Zullo et al., 2019). Intestinal DAF-16/FOXO activation alone substantially promotes longevity in *daf-16*/*FOXO* and *daf-2* double mutants, indicating that the intestine also acts as a central longevity-regulating tissue that communicates with other tissues (Libina et al., 2003, Zhang et al., 2022). Intestinal DAF-16/FOXO coordinates systemic IIS activity through negative feedback regulation of INS-7, which transmits inhibitory signals from the intestine to neurons to attenuate neuronal IIS and synchronize DAF-16/FOXO activity across tissues (Murphy et al., 2003, Murphy et al., 2007). Intestinal AQP-1/aquaporin (AQP3/AQP7/AQP9), a DAF-16/FOXO target, downregulates *ins-7* to attenuate neuronal IIS, thereby reinforcing bidirectional coordination between neuronal and intestinal IIS activities (Lee et al., 2009). In addition, INS-6, another ILP that is secreted from ASI and ASJ sensory neurons, decreases intestinal DAF-16/FOXO activity, shortening lifespan (Artan et al., 2016). The role of bidirectional regulation of neuronal and intestinal IIS in promoting longevity is confirmed by the finding that neuronal *daf-2* deficiency activates intestinal DAF-16/FOXO and intestinal *daf-2* deficiency activates neuronal DAF-16/FOXO (Uno et al., 2021), consistent with key earlier findings (Libina et al., 2003; Wolkow et al., 2000). Conversely, acute activation of the neuronal tyramine release activates intestinal tyramine receptor 3 (TYRA-3) and IIS, preventing the nuclear translocation of DAF-16/FOXO and suppressing stress response and longevity (De Rosa et al., 2019). This inter-tissue communication is essential for systemic longevity regulation in *daf-2* mutants. The Notch ligand *dos-3*/delta-like noncanonical Notch ligand 1 *(DLK1)* is a secreted transcriptional target of DAF-16/FOXO that maintains germline stem/progenitor cells by activating Notch signaling in the germline (Zhang et al., 2024). Together, these studies establish that the IIS pathway is not a linear signaling cascade but a complex network of kinases, transcription factors, and inter-tissue signals.

### Mechanistic Insights Into DR-Mediated Longevity in *C. elegans*

DR, defined as reduced nutrient intake without malnutrition, is a conserved intervention that extends lifespan across species from yeast to mammals (Fontana et al., 2010, Green et al., 2022). In *C. elegans*, multiple DR paradigms, including bacterial dilution, axenic culture, intermittent fasting, peptone dilution, bacterial food deprivation, and the genetic DR-mimetic *eat-2* mutations, extend lifespan through both overlapping and independent genetic mechanisms (Greer and Brunet, 2009) (Fig. 2A). The PHA-4/Forkhead box A (FOXA) is necessary for lifespan extension induced by bacterial dilution and *eat-2* mutations (Panowski et al., 2007). HSF-1/HSF1 contributes to longevity induced by bacterial food deprivation, but is dispensable for longevity caused by *eat-2* mutations (Hsu et al., 2003; Steinkraus et al., 2008). These findings indicate that DR is not a uniform molecular condition but a continuum of nutrient-limited states that elicit distinct transcriptional and metabolic responses. Despite these differences, many DR paradigms converge on conserved nutrient-sensing pathways such as IIS, mechanistic target of rapamycin (mTOR), AAK-2/AMP-activated protein kinase (AMPK), and GCN-2/eukaryotic translation initiation factor 2 alpha kinase 4 (EIF2AK4), which remodel metabolism, enhance autophagy, and increase stress resistance (Greer et al., 2007; Hansen et al., 2008; Rousakis et al., 2013). Thus, DR promotes longevity through evolutionarily conserved mechanisms linking nutrient availability to metabolic adaptation and cellular maintenance (Fontana et al., 2010, Green et al., 2022) (Fig. 2B).

Distinct DR paradigms engage these conserved pathways through different mechanisms. Under axenic dietary restriction (ADR), in which animals are cultured in sterile medium containing essential nutrients without bacterial food sources, *C. elegans* exhibit an extended lifespan (Houthoofd et al., 2002), although improvements in healthspan are limited (Wu et al., 2024). The BLI-4/proprotein convertase subtilisin/kexin (PCSK) acts in neurons and is essential for ADR-mediated longevity (Wu et al., 2025). Loss of BLI-4/PCSK abrogates ADR-mediated lifespan extension and reduces the Golgi factor GOLG-2/Golgin subfamily A member 2 (GOLGA2), implicating secretory-pathway control in this program (Wu et al., 2025). Intermittent fasting represents another DR paradigm (Honjoh et al., 2009). Intermittent fasting requires RHEB-1/Ras homolog enriched in brain (RHEB)-TOR signaling to promote longevity, as RHEB-1/RHEB is essential for fasting-triggered DAF-16/FOXO activation and the transcriptional response to fasting (Honjoh et al., 2009). Periodic fasting and refeeding cycles extend lifespan by activating DAF-16/FOXO and the transcription factor activator protein 1 (AP-1), composed of JUN-1/Jun proto-oncogene (JUN) and FOS-1/FBJ osteosarcoma oncogene (FOS) (Uno et al., 2013). AP-1 enhances ubiquitin-dependent proteostasis through the JNK pathway (Ahn et al., 2023; Uno et al., 2013). In addition, the miRNA-processing enzyme DRSH-1/drosha ribonuclease III (DROSHA) mediates intermittent fasting-derived longevity by increasing the expression of DAF-16/FOXO target genes (Kogure et al., 2017). Intermittent fasting also extends lifespan and improves oxidative stress resistance in a miR-34-dependent manner (Yang et al., 2013). Together, these findings demonstrate that ADR and intermittent fasting increase lifespan by modulating stress response, nutrient sensing, and gene expression.

A well-established genetic DR model is the *eat-2* mutants, in which defective pharyngeal nicotinic acetylcholine receptors reduce pumping and food intake (Lakowski and Hekimi, 1998). The *eat-2* mutations extend lifespan through DR-related nutrient-sensing pathways and are accompanied by enhanced intestinal autophagy and lysosomal activity, which is essential for longevity (Gelino et al., 2016, Hansen et al., 2008, Sun et al., 2020). ZIP-2/bZIP transcription factor family 2, which is crucial for the innate immune response, is activated in *eat-2* mutants and contributes to improved mitochondrial integrity and physical performance (Hahm et al., 2019). These findings and many other studies that we were not able to cover here establish *eat-2* mutations as a genetic DR model that promotes longevity through enhanced autophagy, lysosomal activity, and immune defense.

Different nutrient components also determine DR outcomes. Glucose restriction promotes longevity through neuronal AAK-2/AMPK isoform-dependent lipid remodeling (Jeong et al., 2023). Conversely, excessive amount of glucose shortens lifespan by inhibiting DAF-16/FOXO and HSF-1/HSF1 (Lee et al., 2009). Sensory cues such as food odor can antagonize DR-derived longevity by reducing DAF-16/FOXO activity, thereby linking environmental perception to nutrient-dependent longevity (Park et al., 2021b). The bile acid metabolite lithocholic acid (LCA) is elevated in the serum of calorie-restricted mice, where LCA functions as a DR-derived metabolic signal (Qu et al., 2025a, Qu et al., 2025b). In *C. elegans*, exogenous LCA acts as a DR-mimetic and extends lifespan by activating the conserved AAK-2/AMPK-SIR-2.1/SIRT1 axis through TUB-1/TUB-like protein 3 (TULP3)-dependent signaling (Qu et al., 2025a, Qu et al., 2025b). Another small molecule, C22, extends *C. elegans* lifespan by inducing the DR-responsive gene *fmo-2*/flavin-containing dimethylaniline monooxygenase (*FMO2*/*FMO3*/*FMO4*) and functions in the same pathway as DR (Beydoun et al., 2023). Metformin induces a DR-like metabolic state and increases lifespan and healthspan by acting via AAK-2/AMPK and SKN-1/NRF (Onken and Driscoll, 2010). The longevity effects of metformin depend on dietary, microbial, and age-dependent physiological contexts (Cabreiro et al., 2013, Espada et al., 2020). In *C. elegans*, metformin extends lifespan only with metformin-sensitive *Escherichia coli* by inducing microbial folate and methionine restriction, whereas metformin decreases lifespan under axenic or metformin-resistant bacterial conditions (Cabreiro et al., 2013). Furthermore, treatment with metformin initiated in late life causes mitochondrial dysfunction and ATP exhaustion, leading to short lifespan (Espada et al., 2020). The DR-mimetic effect of metformin is mediated by lysosomal activation of AAK-2/AMPK (Ma et al., 2022). In this pathway, γ-secretase subunit PEN-2/presenilin enhancer (PSENEN) interacts with VHA-19/ATPase H^+^ transporting accessory protein 1 (ATP6AP1), enabling AMPK activation independently of AMP/ADP ratio changes (Ma et al., 2022). Overall, nutrient-specific regulation plays a crucial role in DR-derived lifespan extension.

DR drives a comprehensive metabolic reprogramming that optimizes energy utilization and preserves proteostasis to promote longevity (Anderson and Weindruch, 2010). DR remodels cellular metabolism by decreasing rRNA synthesis and anabolic processes such as protein and lipid biosynthesis, thereby reducing energy consumption and enhancing proteostasis (Rollins et al., 2019; Tiku et al., 2017). Inhibition of rRNA synthesis remodels lipid metabolism toward increased monounsaturated fatty acid levels and triacylglycerol retention, reducing energetic and translational burden (Sharifi et al., 2024). ACS-20/acyl-CoA synthases/fatty acid transport protein 4 (FATP4) is an essential mediator of DR-derived longevity and healthspan (Wang et al., 2023). ACS-20/FATP4 acts in the epidermis via NHR-23/RAR-related orphan receptor A (RORA) to repress PTR-8/patched domain containing 1 (PTCHD1), linking tissue-specific lipid metabolic status to maintenance of proteostasis under nutrient-limiting conditions (Wang et al., 2023). DR initiated at the early adult stage is most effective for lifespan extension, as young animals exhibit higher metabolic flexibility (Loo et al., 2024). Mild DR enhances β-oxidation and suppresses N-acetyl-L-methionine and S-adenosyl-methionine metabolism to promote autophagy and maintain cellular homeostasis (Loo et al., 2024). Unlike DR, glucose-rich conditions shorten lifespan, whereas LPIN-1/lipin 1 (LPIN1) preserves ω-6 polyunsaturated fatty acid levels to counteract the detrimental effects of glucose-induced lipid imbalance and lifespan shortening (Jung et al., 2020). The level of phosphocholine, a key intermediate in the phosphocholine biosynthesis pathway that accumulates with age, is decreased in *eat-2* mutants (Pontoizeau et al., 2014). Under DR, increased autophagic activity, improved lysosomal capacity, and epigenetic remodeling contribute to proteostasis maintenance (Ji et al., 2024; Lee et al., 2024; Lim et al., 2023; Matai et al., 2019). Lysosome also acts as metabolic signaling hubs under nutrient-limiting conditions, where starvation-induced lipolysis and lysosomal AAK-2/AMPK activation promotes histone H3.3 methylation to sustain long-term proteostasis and longevity across generations (Zhang et al., 2025). In *eat-2* mutants, longevity depends on maintaining low mitochondrial permeability, which preserves autophagy-mediated proteostasis (Zhou et al., 2019). Thus, DR promotes longevity by reprogramming cellular metabolism to efficient energy utilization and enhanced proteostasis.

Trade-offs are an intrinsic feature of DR. While extending lifespan, DR delays development and reduces adult body size and reproductive capacity (Crawford et al., 2007, Lee et al., 2016). These phenotypes reflect a reallocation of resources from growth and reproduction to somatic maintenance under DR. Temporary fasting-induced longevity in *C. elegans* increases mortality and reduces reproductive fitness in great-grand descendants (F3 generation), indicating transgenerational trade-offs associated with DR-mediated lifespan extension in P_0_ animals (Ivimey-Cook et al., 2021). At the cellular and molecular levels, DR suppresses IIS and mTOR signaling, thereby reducing protein synthesis and germ cell formation, while promoting longevity through DAF-16/FOXO activation (Greer et al., 2007, Korta et al., 2012). Consistent with this resource shift, partial inhibition of RNA polymerase III, a downstream effector of mTOR complex 1 (mTORC1), extends lifespan in *C. elegans* and *Drosophila* by reducing protein synthesis and improving proteostatic stress tolerance, thereby mimicking DR (Filer et al., 2017). Depletion of *mel-26*/kelch-like family member 22 (*KLHL22*) extends lifespan by maintaining amino acid-dependent mTORC1 inhibition (Chen et al., 2018). DR also preserves pre-mRNA splicing fidelity through the SFA-1/splicing factor 1 (SF1), which is essential for longevity conferred by both DR and inhibition of mTORC1 components such as RAGA-1/Ras-related GTP-binding A (RRAGA) and RSKS-1/ribosomal protein S6 kinase B2 (RPS6KB2) (Heintz et al., 2017). DR-derived longevity requires fine-tuned suppression of innate immunity (Wu et al., 2019). DR downregulates nutrient-activated p38 mitogen-activated protein kinase (MAPK)-ATF-7/activating transcription factor 7 (ATF7) signaling and innate immune gene expression independently of mTORC1, linking immune suppression to DAF-16/FOXO-mediated longevity (Wu et al., 2019). Overall, DR promotes longevity through metabolic reprogramming and stress-response control, but in many cases at the cost of growth, fertility, and immune defense.

### Mild Inhibition of Mitochondrial Function Extends Lifespan via Coordinated Redox and Mitochondrial Quality Control Networks in *C. elegans*

Mitochondria are central to energy metabolism and redox homeostasis (Lee and Yoon, 2024; Lee et al., 2023; Zong et al., 2024). Paradoxically, reduced mitochondrial activity, which generates mild oxidative stress, represents a conserved longevity regimen across species (Hwang et al., 2012). Reduction-of-function mutations in electron transport chain components, including *clk-1*/coenzyme Q7 (*COQ7*), *isp-1*/ubiquinol-cytochrome c reductase (*UQCRFS1*), and *nuo-6*/NADH:ubiquinone oxidoreductase subunit B4 (*NDUFB4*), decrease mitochondrial respiration and increase lifespan (Dillin et al., 2002; Feng et al., 2001; Lakowski and Hekimi, 1996; Lee et al., 2003; Yang and Hekimi, 2010a). Similar effects have been observed in *Drosophila* and mice, where partial inhibition of mitochondrial respiration extends lifespan and healthspan (Copeland et al., 2009, Liu et al., 2005). These early studies establish the concept that mild mitochondrial dysfunction can promote longevity. Reduced mitochondrial respiration lengthens lifespan through multiple mechanisms, including ROS signaling, activation of the UPR^mt^, metabolic remodeling, and selective clearance of damaged organelles through mitophagy (Guo and Chiang, 2022; Hwang et al., 2012) (Fig. 3).

The central mechanism underlying this longevity involves altered redox signaling. While excessive ROS cause cellular damage, mild increases in ROS can stimulate HIF-1/hypoxia-inducible factor-1α (HIF-1α) to promote longevity (Lee et al., 2010). Transient elevation of mitochondrial ROS during larval development imprints a long-lasting redox state and extends lifespan by reducing global histone H3 lysine 4 trimethylation levels through redox-sensitive inhibition of methyltransferase SET-2/SET domain containing 1A (SETD1A) (Bazopoulou et al., 2019). Elevated mitochondrial ROS activate DAF-16/FOXO to promote longevity in *clk-1*/*COQ7*, *isp-1*/*UQCRFS1*, and *nuo-6*/*NDUFB4* mutants, suggesting that DAF-16/FOXO is a key downstream transcription factor of longevity in multiple pathways (Senchuk et al., 2018). In addition, mitochondrial ROS extend lifespan by activating a feedback loop between HIF-1/HIF-1α and AAK-2/AMPK (Hwang et al., 2014). HIF-1/HIF-1α amplifies ROS to enhance immune and stress responses, while AMPK counteracts excessive ROS to maintain redox balance (Hwang et al., 2014). Consistently, treatment with antioxidants suppresses the extended lifespan of *isp-1*/*UQCRFS1* and *nuo-6*/*NDUFB4* mutants (Yang and Hekimi, 2010b). Low doses of mitochondrial inhibitors such as rotenone significantly extend lifespan by mildly elevating ROS levels, inhibiting mitochondrial ATP synthesis, and activating endogenous antioxidant defenses (Schmeisser et al., 2013). Similarly, a pharmacological screen identified several mitochondrial ATP synthesis inhibitors that promote oxidative stress resistance and extend lifespan through *sod-3*/superoxide dismutase (*SOD3*) upregulation (Ikeda et al., 2020). VRK-1/vaccinia virus-related kinase 1 (VRK1), a serine/threonine kinase involved in energy stress responses, activates AAK-2/AMPK through phosphorylation to promote lifespan extension in respiration mutants (Park et al., 2020). Collectively, these findings indicate that partial inhibition of mitochondrial respiration delays aging by balancing energy production with redox signaling.

Mitochondrial quality control pathways are also crucial for longevity (Moehle et al., 2019, Kim et al., 2023b). The UPR^mt^ enhances proteostasis when mitochondrial function is impaired and is required for the longevity of *clk-1*/*COQ7* and *isp-1*/*UQCRFS1* mutants (Durieux et al., 2011, Houtkooper et al., 2013, Wu et al., 2018). ATFS-1/activating transcription factor 5 (ATF5), a key transcription factor in UPR^mt^, accumulates in the nucleus to activate chaperones and proteases under mitochondrial stress conditions (Nargund et al., 2012). Upon mild mitochondrial stress, ATFS-1/ATF5 upregulates innate immunity genes through the p38 signaling pathway to enhance antioxidant defense and proteostasis (Campos et al., 2021, Kim et al., 2023a). ATFS-1/ATF5 further promotes efficient mitochondrial protein import by regulating import machinery components, thereby contributing to mitochondrial proteostasis and longevity (Xin et al., 2022). Retromer-mediated Wnt signaling through EGL-20/Wnt family member 16 (WNT16) promotes cell nonautonomous ATFS-1/ATF5 activation, coordinating systemic mitochondrial stress response (Zhang et al., 2018). The polysaccharide colanic acid in bacterial food acts directly on *C. elegans* intestinal mitochondria to increase mitochondrial fragmentation and to activate ATFS-1/ATF5, thereby extending lifespan (Han et al., 2017). Histone demethylases Jumonji domain protein 1.2 (JMJD-1.2)/PHD finger protein 8 (PHF8) and JMJD-3.1/JMJD3 increase ATFS-1/ATF5-dependent UPR^mt^ activation and extend lifespan in response to mitochondrial stress (Merkwirth et al., 2016). Neuronal mitochondrial stress activates ATFS-1/ATF5-dependent UPR^mt^ in distal tissues through serotonin signaling, promoting systemic proteostasis and longevity (Berendzen et al., 2016). Similarly, mitochondrial translation defects or pharmacological inhibition of mitochondrial translation activate ATFS-1/ATF5-dependent UPR^mt^ and extend lifespan (Guo et al., 2023).

In addition to ATFS-1/ATF5-dependent UPR^mt^, several pathways reinforce mitochondrial quality control to maintain proteostasis and promote longevity. Knockdown of mitochondrial chaperone *hsp-6*/heat shock protein family A (Hsp70) member 9 (*HSPA9*) disrupts mitochondrial proteostasis but triggers a compensatory mitochondrial-to-cytosolic stress response (Kim et al., 2016). This adaptive response remodels lipid metabolism and activates the UPR^mt^, enhancing cytosolic proteostasis and potentially promoting longevity (Kim et al., 2016). Inhibition of mitochondrial translation via knockdown of *mrps-5*/mitochondrial ribosomal protein S5 (*MRPS5*) extends lifespan through an immunometabolic stress response mediated by C32E8.9/ethylmalonyl-CoA decarboxylase 1 (ECHDC1) (Hu et al., 2025). This pathway activates TGF-β/SMA-4/SMAD family member 4 (SMAD4)-dependent innate immunity and lipid remodeling independently of the UPR^mt^ (Hu et al., 2025). Collectively, these studies elucidate how mitochondrial quality control integrates organelle communication and immune response to maintain intracellular homeostasis and to extend lifespan.

Mitochondria-to-nucleus signaling plays a pivotal role in coupling mitochondrial stress to nuclear transcriptional adaptation. Mild mitochondrial stress decreases nuclear pore complex transport, thereby inhibiting mTORC1 and extending lifespan (Wu et al., 2016). Reduced acetyl-coenzyme A signals mitochondrial stress to activate nucleosome remodeling and NuRD/deacetylase-dependent repressive chromatin remodeling that promotes longevity (Park et al., 2023; Zhu et al., 2020). Disruption of NuRD components suppresses longevity conferred by mild mitochondrial dysfunction by downregulating UPR^mt^ (Zhu et al., 2020). Mild mitochondrial stress during development triggers widespread chromatin reorganization through MET-2/SET domain bifurcated histone lysine methyltransferase 1 (SETDB1)-dependent histone H3 lysine 9 (H3K9) dimethylation (H3K9me2) (Tian et al., 2016). This stress also increases abnormal cell lineage 65 (LIN-65) nuclear accumulation (Tian et al., 2016). These changes facilitate DVE-1/SATB homeobox (SATB1/SATB2) recruitment and activation of the UPR^mt^, promoting mild mitochondrial stress-derived longevity (Tian et al., 2016). Epigenetic repressors BAZ-2/bromodomain adjacent to zinc finger domain 2B (BAZ2B) and SET-6/euchromatic histone lysine methyltransferase 1 (EHMT1) decrease mitochondrial gene expression through H3K9me3, thereby reducing mitochondrial function (Yuan et al., 2020). This reduction facilitates UPR^mt^ activation and improves healthspan in an ATFS-1/ATF5- and UBL-5/ubiquitin like 5 (UBL5)-dependent manner (Yuan et al., 2020). Together, mitochondria-to-nucleus signaling integrates metabolic and epigenetic cues to remodel chromatin, activates stress-responsive transcriptional programs, and promotes longevity under mild mitochondrial dysfunction.

Mitophagy is another critical quality control process that eliminates damaged mitochondria to maintain cellular homeostasis. Loss of key mitophagy regulators, including *pink-1*/PTEN-induced kinase 1 (*PINK1*) and the mitophagy receptor *dct-1*/BCL-2-interacting protein 3 (*BNIP3*), suppresses the extended lifespan of *isp-1*/*UQCRFS1* mutants (Palikaras et al., 2015). Under reduced mitochondrial respiration conditions, *dct-1*/*BNIP3* is transcriptionally upregulated by SKN-1/NRF, linking mitochondrial quality control to stress resistance (Palikaras et al., 2015). These findings indicate that reduced mitochondrial function is beneficial when coupled with efficient organelle clearance. Urolithin A, a natural mitophagy activator, extends lifespan by preventing the accumulation of aged mitochondria and improving locomotion and pharyngeal pumping in *C. elegans* (Ryu et al., 2016). These effects depend on SKN-1/NRF, and core autophagy factors (Roussos et al., 2025). Tomatidine also requires both DCT-1/BNIP3 and PINK-1/PINK1 to improve healthspan and lifespan (Fang et al., 2017), suggesting that reduced mitochondrial function effectively extends lifespan when coupled with active mitophagy. Beyond classical mitophagy, communication between mitochondria and Golgi apparatus also contributes to mitochondrial quality control. The Golgi protein MON-2/monensin sensitivity homolog 2 (MON2) is upregulated in respiration mutants and promotes longevity by increasing autophagy through LGG-1/gamma-aminobutyric acid receptor-associated protein (GABARAP) activation, highlighting the importance of organelle crosstalk in maintaining mitochondrial homeostasis (Jung et al., 2021, Kim et al., 2024). HPO-27/maestro heat-like repeat family member 1 (MROH1), which promotes lysosomal fission to maintain organelle homeostasis, is essential for mitochondrial quality control (Li et al., 2024a). Loss of *hpo-27*/*MROH1* disrupts communication between mitochondria and lysosomes, and suppresses the extended lifespan of *isp-1*/*UQCRFS1* and *eat-2* mutants (Li et al., 2024a). Enhancing mitochondrial proteostasis through coordinated activation of the UPR^mt^ and mitophagy also promotes longevity (Sorrentino et al., 2017). In a *C. elegans* Aβ proteotoxicity model, pharmacological or genetic activation of the mitochondrial stress response improves proteostasis, reduces amyloid-β aggregation, and extends lifespan, and these benefits require the UPR^mt^ regulator a*tfs-1*/*ATF5* and the mitophagy receptor *dct-1*/*BNIP3* (Sorrentino et al., 2017). Collectively, reduced mitochondrial function promotes longevity by eliciting adaptive redox signaling, activating mitochondrial stress responses, and maintaining mitochondrial quality control.

### Germline Deficiency Links Reproductive Status to Systemic Longevity Regulation in *C. elegans*

The reproductive system plays a major role in aging in *C. elegans* and serves as a key example of tissue-to-tissue communication in lifespan regulation (Antebi, 2013; Hsin and Kenyon, 1999; Lee et al., 2015b). Removal of germline stem cells extends lifespan, whereas removal of both the germline and somatic gonad does not (Antebi, 2013; Hsin and Kenyon, 1999; Lee et al., 2015b). The temperature-sensitive germline proliferation 1 (*glp-1*) mutants, the most widely used genetic model of germline deficiency, display lifespan extension at a restrictive temperature (25ºC) (Arantes-Oliveira et al., 2002). Hermaphrodites undergo a self-destructive reproductive program that converts somatic resources into yolk, accelerating aging, while germline ablation suppresses this process and markedly extends lifespan (Ezcurra et al., 2018, Kern et al., 2023). Similar longevity effects of germline removal have been observed in *Drosophila* and potentially in humans, indicating that reproductive regulation of aging is evolutionarily conserved (Flatt et al., 2008, Min et al., 2012). Together, these findings indicate that germline signaling suppresses longevity, and the somatic gonad is required for germline-loss-induced lifespan extension.

Germline deficiency extends lifespan by activating endocrine and transcriptional networks that link reproduction to somatic maintenance and stress resistance (Antebi, 2013) (Fig. 4). Upon germline ablation, the nuclear receptor DAF-12/vitamin D receptor (VDR) and the transcription factor DAF-16/FOXO are activated to initiate somatic longevity programs (Antebi, 2013; Hsin and Kenyon, 1999). DAF-12/VDR is activated by dafachronic acids (DAs) synthesized by the cytochrome P450 enzyme DAF-9/cytochrome P450 family 27 subfamily A member 1 (CYP27A1) (Antebi, 2013). DAF-16/FOXO requires the intestinal KRI-1/Krev interaction trapped/cerebral cavernous malformation 1 (KRIT1) for nuclear localization in germline-deficient animals, although KRI-1/KRIT1 does not itself act as the upstream signal that triggers DAF-16/FOXO activation (Berman and Kenyon, 2006, Wei and Kenyon, 2016). DAF-16/FOXO and DAF-12/VDR cooperatively induce lipid-metabolic genes such as lipase-related 17 (*lips-17*) and *fard-1*/fatty acyl-CoA reductase 1 (*FAR1*), both of which are required for germline-loss-induced longevity (McCormick et al., 2012). *tcer-1*/transcription elongation regulator 1 (*TCERG1*) is upregulated upon germline removal and is essential for the induction of DAF-16/FOXO target genes that promote longevity in germline-deficient animals (Ghazi et al., 2009). The transcription factor HLH-30/TFEB is activated in germline-deficient animals and increases the expression of autophagy- and lysosome-associated genes required for metabolic remodeling and longevity upon germline loss (Lapierre et al., 2013). This transcriptional remodeling links reduced reproductive activity to enhanced somatic maintenance.

Germline removal remodels somatic lipid metabolism to support enhanced maintenance (Pires da Silva et al., 2024). The nuclear receptor NHR-49/peroxisome proliferator-activated receptor α (PPARα) (Woo et al., 2024) is transcriptionally upregulated by DAF-16/FOXO upon germline loss and promotes fatty acid β-oxidation, contributing to lifespan extension in germline-deficient animals (Ratnappan et al., 2014). Germline-deficient animals accumulate excess fat due to unconsumed yolk, and the resulting unsaturated fatty acid signals activate SKN-1/NRF, which increases fatty acid oxidation that balances fat storage and mediates lifespan extension (Steinbaugh et al., 2015). Germline loss increases both lipid anabolic and catabolic pathways to maintain lipid homeostasis, and this adaptation is driven by DAF-16/FOXO and TCER-1/TCERG1, which upregulate genes for lipid synthesis and breakdown that contribute to longevity (Amrit et al., 2016). Germline ablation activates NHR-80/hepatocyte nuclear factor 4 (HNF4), which upregulates *fat-6*/stearoyl-CoA desaturase (*SCD*) to increase oleic acid levels, and this fatty acid desaturation pathway is essential for germline deficiency-derived longevity (Goudeau et al., 2011). Autophagy modulates lipid metabolism to mediate longevity in germline-deficient mutants (Lapierre et al., 2012). Autophagy and the triacylglycerol lipase LIPL-4/lipase family member J and K (LIPJ/LIPK) contribute to longevity interdependently in germline-deficient animals (Lapierre et al., 2011). Autophagy is required to maintain high LIPL-4/LIPJ/LIPK-dependent lipase activity, which is required for increased autophagy, contributing to lipid homeostasis.

Germline deficiency affects diverse signaling axes that connect reproductive status with systemic physiology. Loss of germline cells reduces cadherin-mediated adhesion between the distal tip cell and germline stem cells, and this reduction in gonadal adhesion contributes to lifespan extension (Liu et al., 2024). Germline-specific knockdown of the DIMT-1/DIM1 rRNA methyltransferase and ribosome maturation factor (DIMT1), which catalyzes N6-dimethyladenosine methylation on 18S rRNA, extends lifespan (Rothi et al., 2025). Depletion of DIMT-1/DIMT1 changes selective ribosome binding to specific mRNAs in the germline, including a reduction in ribosome occupancy on *daf-9*/*CYP27A1* mRNA, modifying germline-to-soma signaling to promote longevity (Rothi et al., 2025). Transcriptomic analyses demonstrate that germline deficiency increases lifespan and substantially decreases lifespan variance, indicating that the germline generates inter-individual variability in aging (Eder et al., 2024). Germline loss preserves somatic proteostasis in early adulthood, including heat shock response activity, metastable protein folding, and resistance to polyQ aggregation, through various longevity-promoting factors (Shemesh et al., 2013). Germline loss also enhances innate immunity. Germline removal increases the expression of infection response gene 7 (*irg-7*), which activates the p38 MAPK/ATF-7/ATF7 innate immune pathway for enhancing pathogen resistance and is required for longevity in glp-1 mutants (Yunger et al., 2017). In addition, NHR-49/PPARα in germline-deficient animals upregulates innate immunity genes and enhances pathogen resistance (Naim et al., 2021). Collectively, these findings highlight that germline deficiency activates hormonal, transcriptional, and cell-cell communication pathways that couple reproductive status to systemic metabolism and stress responses, thereby promoting longevity.

## CONCLUSIONS AND PERSPECTIVES

Longevity in *C. elegans* is regulated by evolutionarily conserved pathways that include reduced IIS, DR, mild inhibition of mitochondrial respiration, and germline deficiency (Kenyon, 2010, Lee et al., 2015b). Although these pathways originate from distinct upstream cues, they converge on common downstream processes that reinforce proteostasis, RNA homeostasis, metabolic remodeling, and stress resistance to promote longevity (Guo et al., 2022; Kwon et al., 2023; Kuzu et al., 2025; Ottens et al., 2021). Transcriptional regulators such as DAF-16/FOXO, PHA-4/(FOXA), SKN-1/NRF, HLH-30/TFEB, and ATFS-1/ATF5 coordinate these adaptive responses to maintain cellular integrity and delay functional declines during aging (Chae et al., 2023; Denzel et al., 2019; Kenyon, 2010; Nakamura and Yoshimori, 2018).

Despite extensive advances, the molecular and physiological connections among these longevity pathways remain incompletely understood. Future studies need to define how pathway-specific regulators cooperate to maintain DNA, RNA, and protein quality control and how inter-tissue signaling networks integrate neuronal, intestinal, and reproductive inputs to coordinate systemic lifespan regulation. It will be also important to determine how mitochondrial redox signaling, chromatin remodeling, and metabolic state intersect to balance cellular stress adaptation with resource allocation during aging.

The principles uncovered in *C. elegans* provide a conceptual framework for understanding conserved longevity mechanisms across species. Many genes and processes involved in IIS, DR, mitochondrial quality control, and reproductive signaling are evolutionarily conserved and contribute to age-associated pathologies in mammals. Elucidating these shared mechanisms will facilitate the development of interventions that enhance healthspan by stabilizing RNA and protein homeostasis, preserving metabolic flexibility, and promoting stress resilience without compromising reproductive capacity.

Although *C. elegans* cannot fully capture mammalian aging processes such as cellular senescence or immune aging, *C. elegans* remains highly effective for identifying conserved genes and pathways. Integrating *C. elegans* genetics with mammalian cellular models, organoids, and *in vivo* validation provides a logical path to dissect species-specific differences and common mechanistic features regarding the regulation of aging and longevity.

## Funding and Support

This work was supported by the National Research Foundation of Korea grant funded by the Korea government (MSIT) (RS-2024-00408712) to S.J.V.L.

## Author Contributions

Dajeong Bong, Hyunwoo C. Kwon, and Seung-Jae V. Lee wrote the paper.

## Declaration of Generative AI and AI-Assisted Technologies in the Writing Process

During the preparation of this work, the authors used Chat GPT 5 (OpenAI) in order to improve language clarity of the paper. After using this tool, the authors reviewed and edited the content as needed and take full responsibility for the content of the publication.

## Declaration of Competing Interest

The authors declare that they have no known competing financial interests or personal relationships that could have appeared to influence the work reported in this paper.
